# Supplementary material for: FERN – a Java framework for stochastic simulation and evaluation of reaction networks
Source: BMC Bioinformatics. 2008 Aug 29;9:356. doi: 10.1186/1471-2105-9-356 (PMC2553347; doi:10.1186/1471-2105-9-356)
Supplement: Additional file 1 — FERN distribution, Version 1.3. This archive contains the FERN source code and binaries as well as documentation and example models in FernML and SBML. [file 1471-2105-9-356-S1.zip › fern/doc/javadoc/fern/tools/Stochastics.html]

Stochastics


---


|  |  |  |
| --- | --- | --- |
| **Overview** \| **Package** \| **Class** \| **Use** \| **Tree** \| **Deprecated** \| **Index** \| **Help** | |  |
| **PREV CLASS**   NEXT CLASS | **FRAMES**    **NO FRAMES**     **All Classes** |  |
| SUMMARY: NESTED \| FIELD \| CONSTR \| METHOD | DETAIL: FIELD \| CONSTR \| METHOD |  |


---


## fern.tools Class Stochastics

```
java.lang.Object
  fern.tools.Stochastics
```

---

``` public class Stochastics extends Object ```

Manages a random number generators for various distributions. By only using this class for every
random number drawing in the whole framework, some advantages emerge:

- it is guaranteed that the most quickest possible generator is used for each drawing
- one can derandomize the algorithms by using `setSeed(Date)`
- the number of random number generations can easily be counted

**Author:**
:   Florian Erhard

---

| **Method Summary** | |
| --- | --- |
| `int` | `getBinom(int i, double prob)`             Gets a random number from the binomial distribution. |
| `int[]` | `getCounts()`             Gets the number of random number generations for each distribution (same order as in `getNames()` since the last call of `resetCounts`. |
| `double` | `getExponential(double d)`             Gets a random number from the exponential distribution. |
| `static Stochastics` | `getInstance()`             Singleton pattern method. |
| `String[]` | `getNames()`             Gets the names of the built-in distributions. |
| `double` | `getNormal()`             Gets a random number from the Normal distribution |
| `double` | `getNormal(double mean, double stddev)`             Gets a random number from the Normal distribution with given mean and stddev. |
| `int` | `getPoisson(double mean)`             Gets a random number from the Poisson distribution. |
| `long` | `getSeed()`             Gets the actual seed of the random number generator. |
| `double` | `getUnif()`             Gets a random number from the uniform distribution between 0 and 1. |
| `int` | `getUnif(int min, int max)`             Gets a uniformly chosen random number between `min` (inclusive) and `max` (exclusive) |
| `boolean` | `isCountGenerations()`             Gets whether or not to count the number of random number generations |
| `void` | `resetCounts()`             Resets the counts for each distribution to zero. |
| `void` | `resetSeed()`             Sets the seed to the current date |
| `void` | `setCountGenerations(boolean countGenerations)`             Sets whether or not to count the number of random number generations |
| `void` | `setSeed(Date seed)`             Sets a new seed for the random number generator. |
| `void` | `setSeed(long seed)`             Sets a new seed for the random number generator. |

**Methods inherited from class java.lang.Object**

`clone, equals, finalize, getClass, hashCode, notify, notifyAll, toString, wait, wait, wait`

**Method Detail**

### getInstance

```
public static Stochastics getInstance()
```

:   Singleton pattern method.

    :   **Returns:**: the singleton object

---


### resetSeed

```
public void resetSeed()
```

:   Sets the seed to the current date

---


### getSeed

```
public long getSeed()
```

:   Gets the actual seed of the random number generator.

    :   **Returns:**: seed

---


### setSeed

```
public void setSeed(long seed)
```

:   Sets a new seed for the random number generator.

    :   **Parameters:**: `seed` - the seed

---


### setSeed

```
public void setSeed(Date seed)
```

:   Sets a new seed for the random number generator.

    :   **Parameters:**: `seed` - the seed

---


### isCountGenerations

```
public boolean isCountGenerations()
```

:   Gets whether or not to count the number of random number generations

    :   **Returns:**: whether or not to count

---


### setCountGenerations

```
public void setCountGenerations(boolean countGenerations)
```

:   Sets whether or not to count the number of random number generations

    :   **Parameters:**: `countGenerations` - whether or not to count

---


### getCounts

```
public int[] getCounts()
```

:   Gets the number of random number generations for each distribution (same order as in
    `getNames()` since the last call of `resetCounts`.

    :   **Returns:**: number of random number generations

---


### getNames

```
public String[] getNames()
```

:   Gets the names of the built-in distributions.

    :   **Returns:**: names of the distributions

---


### resetCounts

```
public void resetCounts()
```

:   Resets the counts for each distribution to zero.

---


### getUnif

```
public double getUnif()
```

:   Gets a random number from the uniform distribution between 0 and 1.

    :   **Returns:**: random number between 0 and 1 **See Also:**: `Uniform.nextDouble()`

---


### getExponential

```
public double getExponential(double d)
```

:   Gets a random number from the exponential distribution.

    :   **Parameters:**: `d` - mean **Returns:**: random number **See Also:**: `Exponential.nextDouble()`

---


### getBinom

```
public int getBinom(int i,
                    double prob)
```

:   Gets a random number from the binomial distribution.

    :   **Parameters:**: `i` - size: `prob` - probability **Returns:**: random number **See Also:**: `Binomial.nextInt(int, double)`

---


### getUnif

```
public int getUnif(int min,
                   int max)
```

:   Gets a uniformly chosen random number between `min` (inclusive) and `max` (exclusive)

    :   **Parameters:**: `min` - inclusive minimum: `max` - exclusive maximum **Returns:**: uniformly chosen random number **See Also:**: `Uniform.nextIntFromTo(int, int)`

---


### getPoisson

```
public int getPoisson(double mean)
```

:   Gets a random number from the Poisson distribution.

    :   **Parameters:**: `mean` - mean **Returns:**: random number **See Also:**: `Poisson.nextInt(double)`

---


### getNormal

```
public double getNormal()
```

:   Gets a random number from the Normal distribution

    :   **Returns:**: random number **See Also:**: `Normal.nextDouble()`

---


### getNormal

```
public double getNormal(double mean,
                        double stddev)
```

:   Gets a random number from the Normal distribution with given mean and stddev.

    :   **Parameters:**: `mean` - the mean: `stddev` - the stddev **Returns:**: random number **See Also:**: `Normal.nextDouble(double, double)`


---


|  |  |  |
| --- | --- | --- |
| **Overview** \| **Package** \| **Class** \| **Use** \| **Tree** \| **Deprecated** \| **Index** \| **Help** | |  |
| **PREV CLASS**   NEXT CLASS | **FRAMES**    **NO FRAMES**     **All Classes** |  |
| SUMMARY: NESTED \| FIELD \| CONSTR \| METHOD | DETAIL: FIELD \| CONSTR \| METHOD |  |


---
